# A Quantified Ginseng (*Panax ginseng* C.A. Meyer*)* Extract Influences Lipid Acquisition and Increases Adiponectin Expression in 3T3-L1 Cells

**DOI:** 10.3390/molecules16010477

**Published:** 2011-01-10

**Authors:** Chia-Rou Yeo, Chen Yang, Ting-Yan Wong, David G. Popovich

**Affiliations:** Department of Chemistry, National University of Singapore, 14 Science Drive 4, Singapore 117543, Singapore

**Keywords:** ginseng, *Panax ginseng*, ginsenosides, adipocytes, adipogenesis, 3T3-L1

## Abstract

A *Panax ginseng* extract (PGE) with a quantified amount of ginsenosides was utilized to investigate its potential to inhibit proliferation, influence lipid acquisition and adiponectin expression in 3T3-L1 cells. Seven fingerprint ginsenosides were quantified using high performance liquid chromatography and their respective molecular weights were further confirmed via LC-ESI-MS analysis from four different extraction methods. Extraction using methanol under reflux produced significantly higher amounts of ginsenosides. The methanol extract consisted of Rg1 (47.40 ± 4.28 mg/g, dry weight of extract), Re (61.62 ± 5.10 mg/g), Rf (6.14 ± 0.28 mg/g), Rb1 (21.73 ± 1.29 mg/g), Rc (78.79 ± 4.15 mg/g), Rb2 (56.80 ± 3.79 mg/g), Rd (5.90 ± 0.41 mg/g). MTT analysis showed that PGE had a concentration-dependent cytotoxic effect on 3T3-L1 preadipocyte and the LC_50_ value was calculated to be 18.2 ± 5 µg/mL. Cell cycle analysis showed minimal changes in all four phases. Differentiating adipocytes treated with ginseng extract had a visible decrease in lipid droplets formation measured by Oil red O staining. Consequently, triglycerides levels in media significantly (*P* < 0.05) decreased by 39.5% and 46.1% when treated at concentrations of1 µg/mL and 10 µg/mL compared to untreated control cells. Western blot analysis showed that the adiponectin protein expression was significantly (*P* < 0.05) increased at 10 µg/mL, but not at 1 µg/mL. A quantified PGE reduced the growth of 3T3-L1 cells, down-regulated lipid accumulation and up-regulated adiponectin expression in the 3T3-L1 adipocyte cell model.

## 1. Introduction

**Figure 1:**
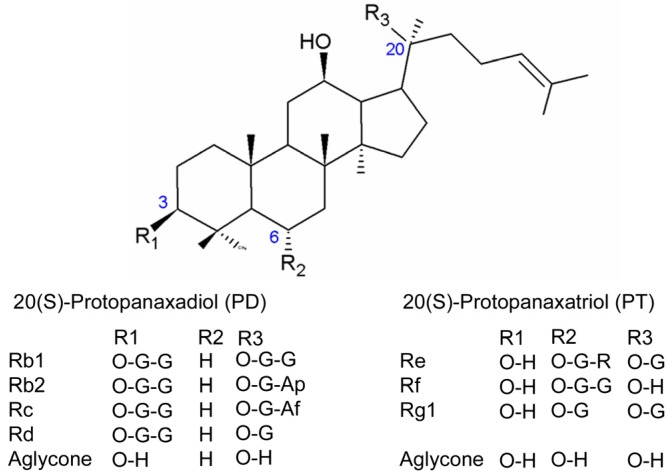
Chemical structure of dammarane type ginsenoside saponins found in the experimental *Panax ginseng* extract (PGE). Regions R1-R3 consists of different attachment of sugar moiety molecules and refer to the following abbreviations: Af: arabinofuranose; Ap: arabinopyranose; G: glucopyranose; R: rhamnopyranose.

Adipocytes play a central role in the regulation of energy balance and mediate numerous factors known to be involved in immunological responses, vascular diseases, and appetite regulation [1,2,3]. An optimal balance in adipocyte regulation is crucial to the maintenance of health and disease prevention and changes in adipocyte number and size often involves a complex interplay between proliferation and differentiation of preadipocytes [2]. Adiponectin, which is secreted from adipocytes, is known to promote adipocyte differentiation, insulin sensitivity and lipid accumulation *in vivo*. Low circulating levels of adiponectin have been associated with metabolic diseases such as the metabolic syndrome and diabetes [4]. Secondary plant metabolites have been reported to influence adipocyte differentiation. For instance, bitter melon (*Momordica charantia*) extract containing both oleanane and dammarane type saponin glycosides [5], red yeast (*Monascus ruber*) rice extract [6], and lanostane triterpenes from the fruiting bodies of *Ganoderma Lucidum* [7,8] have all been shown to influence adipocyte differentiation in cultured 3T3-L1 cells, a murine fibroblast cell line that is often used as a model for adipocyte metabolism. It is noteworthy that the ginseng species is one of the most researched sources of plant saponins. Ginsenosides belong to a diverse group of dammarane triterpenoid glycosides (Figure 1), constructed of four *trans*-ring rigid steroid skeleton with differences in the site and number of attachment of hydroxyl groups [9,10]. Although most of ginsenosides can be classified into two main groups either based on their respective aglycones protopanaxadiol (PD) or protopanaxatriol (PT), individual ginsenosides have been reported to produce different effects on lipid acquisition in 3T3-L1 cells. Huang *et al.* reported that compound K also referred to as ginsenoside CK inhibited triglyceride accumulation in cultured adipocytes while Rg1 enhanced it [11]. Both compounds were shown to activate AMPK (5' AMP-activated protein kinase) and PI3K (phosphatidylinositol 3-kinase) signaling pathways, stimulating glucose uptake in concentration-response manner [11]. Ginsenoside Rb2 cultured under high fatty acid and triglyceride conditions were found to reduce SREBP (sterol regulatory element binding proteins) and stimulate leptin mRNA expression [12]. Ginsenoside Rh2 was shown to have activated glucocorticoid receptor and promoted preadipocytes differentiation [13]. In contrast, ginsenoside Rg3 was shown to have effectively inhibited adipocyte differentiation via AMPK activation and PPARγ (peroxisome proliferator-activated receptor) inhibition [14].The overall pharmacology of ginseng is complex [9] and it is currently unclear if specific ginsenosides or an extract containing ginsenosides is able to exhibit stronger adipocyte metabolism influencing effect. The objective of this study was to demonstrate the effects of Asian ginseng *(Panax ginseng)* extract on preadipocyte cell growth, lipid acquisition and adiponectin expression in 3T3-L1 adipocytes. This study extends our previous work on American ginseng *(Panax quinquefolius)* on 3T3-L1 cells [15]. *Panax ginseng* was extracted using various methods and the respective ginsenoside profiles were compared. The effects on 3T3-L1 cells were assessed by performing cytotoxicity testing on preadipocytes, acquisition of triglyceride levels in media and lipid accumulation status on adipocytes as well as changes in adiponectin expression at the end of 10-day differentiation course.

## 2. Results

### 2.1. HPLC-ESI-MS

**Figure 2:**
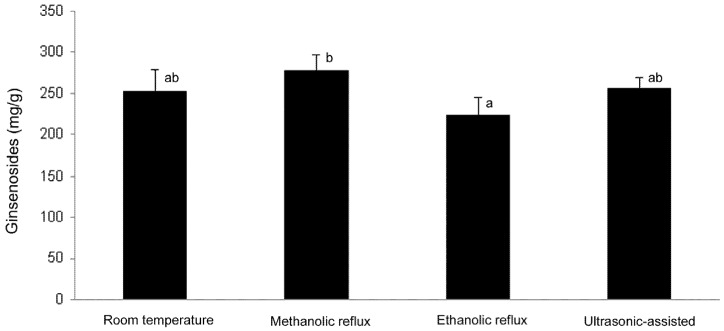
Total ginsenoside content derived from various extraction methods. Columns with a different letter are significantly different (*P* < 0.05).

Ginsenosides profiles generated from different extraction methods were compared and are shown in Figure 2 as summary of all seven ginsenosides and individual ginsenosides in Table 1. Room temperature extraction was heat-free extraction process and it served as a control for statistical comparison. Figure 2 shows the total ginsenosides content derived from room temperature (253.50 ± 26.60 mg/g), methanolic reflux (278.38 ± 19.30 mg/g), ethanolic reflux (224.13 ± 20.75 mg/g), and ultrasonic-assisted (255.89 ± 13.27 mg/g).

**Table 1:** Ginsenoside fingerprint profiles derived from different extraction methods expressed in mg/g. Rows with a different letter are significantly different (*P* < 0.05).

	Room temperature			Methanolic reflux			Ethanolic reflux			Ultrasonic-assisted		
Rg1	48.57	±	2.45	47.40	±	4.28	53.15	±	5.35	51.29	±	3.00
Re	60.94	±	3.99 ^b^	61.62	±	5.10 ^b^	51.04	±	4.60 ^a^	62.98	±	2.60 ^b^
Rf	6.30	±	0.54 ^b^	6.14	±	0.28 ^b^	4.80	±	0.46 ^a^	6.41	±	0.59 ^b^
Rb1	18.79	±	3.89 ^ab^	21.73	±	1.29 ^b^	17.21	±	1.75 ^a^	18.29	±	0.62 ^ab^
Rc	66.43	±	7.56 ^b^	78.79	±	4.15 ^c^	53.47	±	4.80 ^a^	65.73	±	2.95 ^b^
Rb2	48.12	±	7.31 ^ab^	56.80	±	3.79 ^b^	40.54	±	3.38 ^a^	46.82	±	2.95 ^a^
Rd	4.36	±	0.86 ^a^	5.90	±	0.41 ^b^	3.92	±	0.41 ^a^	4.38	±	0.57 ^a^
Total	253.50	±	26.60 ^ab^	278.38	±	19.30 ^b^	224.13	±	20.75 ^a^	255.89	±	13.27 ^ab^

Methanolic reflux extraction significantly (*P* < 0.05) extracted the greatest amount of ginsenosides compared to all other extraction methods. The ethanolic reflux extraction produced a significantly lower (*P* < 0.05) amount of ginsenosides compared to the room temperature control extraction. Individual ginsenosides profile were different depending on the extraction method used (Table 1). Methanolic reflux extraction yielded significantly (*P* < 0.05) higher amount of ginsenosides Rb1, Rc, Rb2 and Rd, while ethanolic reflux extraction generated significantly (*P* < 0.05) lower amount of ginsenosides Re, Rf, Rb1, and Rc. No significant difference was observed for ginsenoside Rg1.

The methanolic reflux extraction produced the highest amount of ginsenosides and contained seven ginsenosides. *Panax ginseng* extract (PGE) were quantified via HPLC analysis using commercial standards. The individual ginsenosides were further assessed using ESI-MS analysis to confirm the molecular weights. Seven ginsenosides were found to produce the most abundant ion as the molecule ion [M − H]^−^ shown in Table 2. The ginsenoside profile consisted of Rg1 (47.40 ± 4.28 mg/g, dry weight of ginseng extract), Re (61.62 ± 5.10 mg/g), Rf (6.14 ± 0.28 mg/g), Rb1 (21.73 ± 1.29 mg/g), Rc (78.79 ± 4.15 mg/g), Rb2 (56.80 ± 3.79 mg/g), Rd (5.90 ± 0.41 mg/g).

**Table 2:** Seven major ginsenosides detected by ESI-MS analysis.

Ginsenosides retention order	Empirical Formula	Molecular weight (Da)	Concentration within extract (mg g^−1^)	Main Ion Fragments, *m/z*	
				[M − H]^−^	Others
Rg1	C_42_H_72_O_14_	801	47.40 ± 4.28	799.6	841.5, 885.3, 927.6
Re	C_48_H_82_O_18_	947	61.62 ± 5.10	945.5	991.4,1110.4,1149.3
Rf	C_42_H_72_O_14_	801	6.14 ± 0.28	799.6	835.5, 1127.4
Rb1	C_54_H_92_O_23_	1109	21.73 ± 1.29	1107.8	1153.3, 1170.5
Rc	C_53_H_90_O_22_	1079	78.79 ± 4.15	1077.6	1123.3,1209.6,1462.7
Rb2	C_53_H_90_O_22_	1079	56.80 ± 3.79	1077.6	1123.4,1150.4, 1209.6
Rd	C_42_H_72_O_14_	947	5.90 ± 0.41	945.6	991.4, 1119.5

### 2.2. MTT LC_50_ Determination and Cell Cycle Distribution

**Figure 3:**
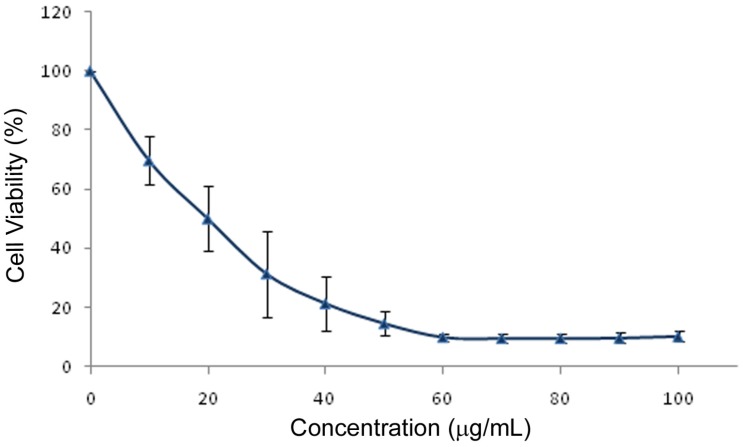
MTT-Dose-response relationship of a ginseng (*Panax ginseng*) extract after 72 h incubation with 3T3-L1 cells assessed by an MTT viability assay. Values are expressed as percentage of untreated control cells (mean ± SD).

The dose-response relationship between PGE and 3T3-L1 preadipocytes growth is shown in Figure 3. LC_50_ was determined from a plot of viability (%) versus log concentration (graph not shown) which yielded a linear equation of y = −81.04*x* + 152.1 (*r*^2^ = 0.992). The LC_50_ was calculated to be 18.2 ± 5 µg/mL.

**Figure 4:**
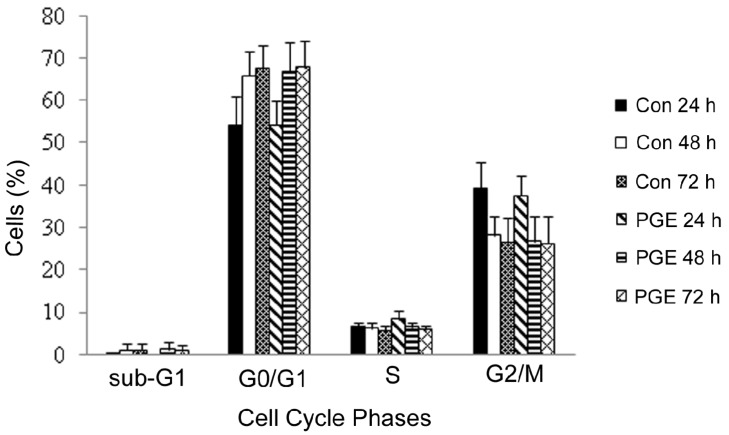
3T3-L1 cells treated with *Panax ginseng* extract (PGE) for 24–72 h. Con refers to untreated control cells. Values are expressed as mean ± SD.

Cell cycle distribution of 3T3-L1 preadipocytes treated with PGE for 24, 48, 72 h, with untreated cells acted as control is shown in Figure 4. Generally, the percentage changes between phases of treated and untreated cells were modest and statistically insignificant. It was observed that there was a slight increase (1.08% and 0.32%) in G0/G1 phase at 48 and 72 h respectively, a slight increase (1.71%, 0.34%, and 0.06%) in S phase and a slight decrease (1.69%, 1.4%, and 0.25%) in G2 phase for PGE-treated cells at 24, 48, 72 h, compared to the untreated controls.

### 2.3. Differentiation, Oil Red O Lipid Staining and Triglycerides

**Figure 5:**
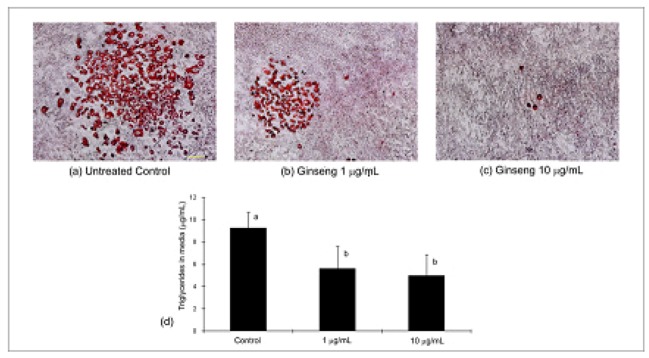
Representative morphological images of lipid uptake in untreated (panel a) and ginseng treated 3T3-L1 cells (panels b, c) and the corresponding quantification of triglycerides in media (panel d). Cells were induced to differentiate as described in the Materials and Methods section. Values are expressed as mean ± SD. Bars with a different letter are significantly different (*P* < 0.05) from each other.

Oil Red O staining of differentiated 3T3-L1 cells is shown in Figure 5. A decrease in the number of lipid droplets formed was observed with increasing concentration of PGE. The amount of triglycerides was significantly (*P* < 0.05) decreased at both concentrations tested. At concentrations of 1 µg/mL and 10 µg/mL, the amount of triglycerides detected in media was 5.60 ± 2.04 µg/mL and 4.99 ± 1.88 µg/mL as compared to untreated control which was 9.26 ± 1.41 µg/mL. There was a significant (*P* < 0.05) decrease of 39.52% and 46.11%, respectively, when compared to control. It is however noteworthy that while the media triglycerides levels between 1 and 10 µg/mL were similar and not found to be significantly different; morphologically, untreated control and cells treated at 1 µg/mL were similar to each other compared to cells treated with 10 µg/mL PGE.

### 2.4. Adiponectin Expression

**Figure 6:**
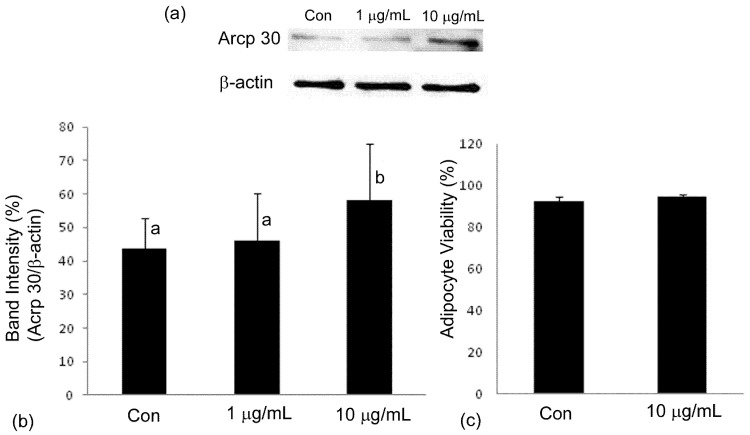
The expression of adiponectin in adipocytes was assessed via Western blot analysis. Panel (a) shows the representative blots while panel (b) shows the densitometer quantification of adiponectin expression (acrp30) in 3T3-L1 adipocytes treated with PGE at 1 µg mL^−1^and 10 µg mL^−1^. Panel (c) shows the viability of the adipocytes at the end of the differentiation process with the highest concentration used for Western blot analysis. Values are expressed as mean ± SD of three independent experiments, as a percentage of β-actin density. Bars with a different letter are significantly different (*P* < 0.05) from each other.

Figure 6 (a,b) show representative Western blots of 3T3-L1 cells which were induced to differentiate and treated with PGE concentrations of 1 and 10 µg/mL, with untreated cells as controls. Adiponectin protein expression significantly (*P* < 0.05) increased with PGE treatment at 10 µg/mL, but not at 1 µg/mL. Figure 6b shows the Western blot band intensities that were calculated utilizing a densitometer and expressed as adiponectin protein bands as percentage of β-actin. At 1 µg/mL, the percentage intensity was 46.0 ± 14.3% while at 10 µg/mL, the % intensity was 58.2 ± 16.8% which was significantly different (*P* < 0.05), compared to control’s intensity value of 43.8 ± 9.0%. It is noteworthy that at a concentration of 10 µg/mL, which was approximately half the LC_50_ value of undifferentiated cells, there was no significant (*P* < 0.05) cytotoxic effect on adipocyte cell growth. Adipocyte viability was assessed using ViaCount dual DNA fluorochrome binding dyes that is capable of distinguishing viable cells from non-viable ones, and is shown in Figure 6c.

## 3. Discussion

Adipocyte differentiation of 3T3-L1 cells is a highly-controlled process that can be induced under a hormonal cocktail of insulin, dexamethasone and IBMX [16,17]. Upon the completion of adipogenesis, preadipocyte fibroblasts that were originally spindle-shaped transform into round-shaped cells that simultaneously accumulate lipids and acquire metabolic mechanism to facilitate glucose uptake in response to insulin, synthesize fatty acids, accumulate triglyceride and secrete a wide variety of hormones and cytokines [1,18]. Therefore, by manipulating adipocyte regulation, the corresponding increase in lipid accumulation and the corresponding risk of obesity may be affected. The intracellular lipid accumulation is commonly monitored as a general marker to indicate the extent of adipogenesis in 3T3-L1 cells [14,19,20]. In this study, we investigated if PGE was able to influence intracellular lipid accumulation in adipocytes and it was found to have reduced it. A significant decrease in triglyceride levels by 39.52% and 46.11% at concentrations of 1 and 10 μg/mL respectively were also observed. Ginsenosides and steroid hormones are very similar in chemical structure due to the steroid skeleton and lipid-solubility. They can then complex cholesterol and permeate cells, likely affecting lipid metabolism [9,21].

Specific ginsenosides and ginseng extract influence on adipocyte regulation have been reported. Both Rb1 and PT aglycone have been shown to promote lipid accumulation and expression of PPARγ and C/EBPα [20,22] both of which are involved in controlling adipocyte differentiation. However research is conflicting, Rb1 was shown to suppress lipid accumulation, suggesting that the period of administration, both during and after differentiation, may lead to different effects [23]. Rb1 and Rg1 suppressed lipid accumulation, down-regulated PPARγ expression and improved insulin sensitivity in 3T3-L1 adipocytes [23]. Minor ginsenosides, Rg3 and Rh2 were shown to have suppressed lipid accumulation, down-regulated the expression of PPARγ and C/EBPα and up-regulated expression of AMPK [14,24]. AMPK is involved in cellular energy regulation. Artificially digested ginsenosides showed inhibitory effects on lipid accumulation in 3T3-L1 adipocytes, with the less polar ginsenoside Rg3 being the most effective [25]. Ginsenoside Rb1 inhibited the proliferation of pre-confluent preadipocytes yet it facilitated the adipogenesis of 3T3-L1 cells [20]. Regarding cell growth, this is parallel to our finding that while PGE displayed cytotoxic effect in preadipocytes, the viability of adipocytes at approximately half the LC_50_ concentration of PGE was not significantly affected. Also, this coincides with a recent report showing that ginsenoside CK and Rg1 had no apparent cytotoxicity at doses up to 10 μM towards differentiated 3T3-L1 cells [11]. The mechanism regarding cell survival is currently unclear. In this study, we suggest that the suppressive effect of the PGE on lipid accumulation was likely to be a combined effect of a cocktail involving many different ginsenosides, which may induce multiple effects. *In vivo*, several studies showed that ginseng powder or ginseng extracts reduced weight gain in animal models [26,27,28,29]. On the contrary, a study using ginseng extract showed anti-lipolytic effect in rat adipocytes mediated by activating PDE4 (phosphodiesterase) in rat adipocytes [30] which contributes to a reduction in inflammation and an influence on cytokine expression. Our results are however consistent with the hypolipidemic effects found in humans. Administration of ginseng extract for eight weeks (6 g per day) decreased serum total cholesterol, triglyceride, LDL, and increased HDL in human subjects [31]. The discrepancies between studies may be inevitable due to the different ginsenoside profile generated from different extracts, as it is known that a single ginsenoside is capable of initiating multiple actions in the same tissue and the overall ginseng pharmacology is complex [9]. At the molecular level, adiponectin expression was significantly (*P* < 0.05) enhanced after treating with PGE extract at 10 μg/mL. Adiponectin is an adipocyte-derived protein that is an important insulin-sensitizing adipocytokine [32,33,34]. Adiponectin exists in a number of forms consisting of homotrimers and multimeric complexes [35]. In serum, adiponectin is found usually as low molecular weight oligomers and high molecular weight multimers [36]. Total adiponectin has been shown to decrease in obesity and diabetes [34] and the high molecular weight form of adiponectin may be of clinical importance [37], although it has not been definitely established which is more relevant. In this study, total adiponectin compromising of both low and high molecular adiponectin were measured to provide evidence of biological activity. Adiponectin increases the expression of proteins involved in fatty acid metabolism, leading to decreased tissue triglyceride content, contributing to improved insulin signal transduction [38]. The differentiating 3T3-L1 cells can be highly heterogeneous in cellular and lipid droplet morphologies, resulting in a mixture of subpopulations reflecting distinct physiological states [39]. There is a highly complex interplay between proliferation and differentiation of adipocytes and preadipocytes, occurring simultaneously [1]. Loo *et al.* suggested that there was only surprisingly small percentage of cells expressing high adiponectin levels and this subpopulation will likely show increased expression of adipogenesis markers [39]. It is noteworthy that subpopulation of quiescent 3T3-L1 cells can also remained throughout the respective observation period. It is currently unknown which subpopulation may be contributing to the adiponectin increase; further studies are needed to address this. These findings provide important insights in knowing that glucose and lipid metabolism of adipocytes treated with ginseng extract were altered even at concentration as low as 10 μg/mL. In our previous work, we have shown that American ginseng *(Panax quinquefolius)* extract exhibited similar effect on 3T3-L1 cells as the Asian ginseng extract PGE. Both extracts reduced cell growth and lipid acquisition, and increase adiponectin expression [15]. Nevertheless, a difference in effective dose was noted. *Panax quinquefolius* extract yielded a LC_50_ value of 40.3 ± 5 μg/mL while with *Panax ginseng* extract the LC_50_ value was 18.2 ± 5 µg/mL. Subsequent experimental design in this present study utilized concentrations that were much lower than that of the previous work and were found to be equally effective, in terms of lipid-lowering activity and adiponectin activation. This suggests that even though both *Panax* species possess similar bioactive constituents, the difference in ginsenoside compositions between the two species could have contributed different pharmacological effects [38]. For example, Asian ginseng was shown to have higher Rg1 to Rb1 ratio compared to American ginseng. It has been shown that Rg1 has a stimulatory effect on the central nervous system, while Rb1 has a weaker or sometimes even suppressive effect [40,41]. In addition, the number and positions of hydroxyl group could influence the pharmacological activity of ginsenosides [42,43]. Ginsenosides appear to be active constituent in ginseng [44]. The specificity of steroidal structure enables them to interact with cell membranes and modulate membrane-bound ion channels, receptors and enzymes, as well as traversing cell membrane producing effects at the genomic level [9]. In particular, the PD aglycone, which differs from the PT aglycone by the absence of one additional hydroxyl group at C-6 position, were shown to be more potent than PT in anti-proliferation of cancer cells as well as anti-obesity effects in an animal model [27,42]. It is noteworthy that total percentage of protopanaxadiol-type (PD) ginsenosides in *Panax quinquefolius* extract was 17.0% while it was 58.6% in *Panax ginseng* extract. *Panax quinquefolius* extract was primarily composed of PT-type ginsenoside Re (79.6%) while in *Panax ginseng* extract Re comprised 22.1% of the total ginsenosides content.

## 4. Experimental

### 4.1. Extraction of Plant Material

Dried Asian ginseng (*Panax ginseng* C.A. Meyer*)* roots were locally purchased, cut into small pieces, ground and extracted using various extraction methods; these included methanolic room temperature extraction, methanolic reflux extraction, methanolic ultrasonic-assisted extraction and ethanolic reflux extraction. Acetonitrile (Tedia, USA), methanol and ethanol (Merck, Germany) used were all of reagent grade and water was purified by Barnstead EasyPure RoDi system (Fisher Scientific, USA).

Methanolic room temperature extraction was carried out in sealed conical flask with stirring. Extraction consisted of 20 g ginseng powder per batch and was conducted for 24 h under room temperature conditions. After 24 h, extract was filtered (Whatman no. 4 paper) and methanol was removed under vacuum and re-suspended in distilled water. A Sigma Amberlite (St. Louis, MO, USA) XAD-4 resin (surface area 725 m^2^/g, pore diameter 40 Ǻ, bed volume of 60 cm^3^ and flow rate of 2 mL/min) column was used for the purification of the extracts [45]. As previously described [46], crude extract was applied to preconditioned XAD-4 resins rinsed with 1 L of distilled water. Ginsenosides were then eluted with 500 mL of absolute ethanol. Similarly, ethanol was removed under vacuum, re-suspended in distilled water, and lyophilized. 

Reflux extraction utilized methanol and ethanol respectively for 4 h each which was repeated three separate times. The extracts were filtered (Whatman no. 4 paper) and solvents were removed under vacuum and re-suspended in distilled water. The extracts were applied to the XAD-4 resin as described above. For ultrasonic- assisted extraction, ginseng powder was thoroughly suspended in methanol and subjected to 4 h of ultrasonication (Elmasonic S30H, Elma GmbH & Co. KG, Singen, Germany). At 2 h interval, extract was filtered once and set aside while the residue was extracted with fresh methanol for another 2 h. Upon completion, both the extracts were combined and solvents removed under vacuum. The extracts were then applied to the XAD-4 as described above. 

Lyophilized product from methanolic room temperature extraction acted as control for the statistical comparison of the extraction methods while the lyophilized product from methanolic reflux extraction was utilized in all subsequent experiments. It is herein referred to as the *Panax ginseng* extract (PGE). 

### 4.2. HPLC and ESI-MS Analysis

To quantify the major ginsenosides content in PGE, a high performance liquid chromatography (HPLC) system (Waters Alliance 2695, Waters, Milford, MA, USA) coupled to a photodiode array detector (Waters 2996) and a control software Empower Pro (Waters) was employed. A Phenomenex (Torrance, CA, USA) reversed phase C-18 column (4.6 mm × 250 mm, 5 μm diameter) was used. The column temperature was set at 25 °C and the sample injection volume was 20 μL. The flow rate was1 mL/min and the detection wavelength was set at 203 nm. The mobile phase consisted of distilled water (A) and acetonitrile (B). The solvent program was as follows at time 0 min, 20% (B); 60 min, 42% (B); 61 min, 90% (B); 71 min, 90% (B); 72 min, 20% (B); 80 min, 20% (B) [47]. 

Ginsenoside standards (Rg1, Re, Rf, Rb1, Rc, Rb2, Rd) were purchased from Chromadex (Santa Ana, CA, USA) and were used to establish calibration curves. The molecular weights of the standards and ginsenosides contained in the PGE were confirmed by LC-MS analysis. A Finnigan-MAT (San Jose, CA, USA) LCQ quadrupole ion trap MS with MS^n^ capabilities in negative mode was employed. The ESI-MS conditions were set as follows: capillary temperature of 250 °C, ion spray voltage of 4.50 kV, capillary voltage of −17 V, sheath gas rate of 80 arbitrary units (arb) and sweep auxiliary gas rate of 20 arb. Sample was delivered to the MS at a flow rate of 0.4 mL/min and scanning mass spectra focused on the *m/z* range of 50–1500 U.

### 4.3. Cell Culture

Murine (3T3-L1) fibroblast cells (preadipocytes), purchased from ATCC (Manassas, VA, USA), were cultured in DMEM (Caisson; Logan, UT, USA) supplemented with 10% fetal bovine serum (Sigma, St Louis MO, USA) and penicillin/streptomycin (100 U/mL) (GIBCO; Invitrogen; Burlington, Canada). The cells were maintained in a humidified atmosphere with 5% CO_2_ at 37 °C. Cells were kept at a concentration between 5 × 10^3^ and 1 × 10^5^ cells/mL. Upon reaching 70% confluence, cells were subcultured in every 3–4 days using 0.25% (w/v) trypsin-0.53 mM EDTA solution (GIBCO). Viable cell numbers were assessed in quadruplicate using a Neubauer hemocytometer (Blaubrand, Germany) and trypan blue (0.04%) exclusion dye (MP Biomedicals, OH, USA). PGE was well dissolved in DMEM and passed through 0.2 μm filter (Millex GP, Ireland) before use.

### 4.4. Cell Viability MTT Assay Dose-Response

To determine the concentration that inhibits 50% of the cells, a dose-response curve was established using the MTT [3-(4,5-dimethylthiazol-2-yl)-2,5-diphenyl tetrazolium bromide] assay. 3T3-L1 preadipocytes were seeded in 96-well plates at a concentration of 2.5 × 10^4^ cells/mL. Controls consisted of cells and media but without PGE. Cells were treated with PGE dissolved in DMEM for a total of 72 h. Media and PGE were removed and MTT solution (0.5 mg/mL dissolved in DMEM) was added and incubated in the dark for 4 h. Sodium dodecyl sulfate (SDS) acidified with 0.01 M HCl was added to dissolve the formazan crystals overnight. On the following day, the optical density was measured at 570 nm with 650 nm as reference absorbance using microplate reader (Multiskan Spectrum, Thermo Electro Corporation, Waltham, MA, USA). Cell viability (%) was calculated by [mean (absorbance of sample at 570 nm – absorbance of reference sample at 650 nm) / mean absorbance of control] × 100%.

### 4.5. Cell Cycle Analysis

Murine 3T3-L1 preadipocytes (2.5 × 10^4 ^cells/mL) seeded in 24-well plates were treated with PGE at its LC_50_ of 18.2 μg/mL (described subsequently) and incubated at 37 °C in a 5% CO_2_ humidified incubator for 24, 48, 72 h, with untreated cells acting as controls. After treatment for 24, 48, 72 h, non-adherent cells in media were collected and centrifuged (10 min, 150 g)*.* Attached cells were trypsinized with 2 mL trypsin for 10 min and mixed with 2 mL of fresh DMEM and centrifuged (10 min, 650 g), and the supernatant were discarded. Both cell pellets from non-adherent and attached cells were washed twice with phosphate buffered saline (PBS) respectively. Cell pellets were combined, vortexed and ice cold 70% ethanol was added slowly to fix the cells and stored overnight at 4 °C. Ethanol was removed by centrifugation (8 min, 500 g) and 1 mL of PBS containing 50 μg/mL propidium iodide (PI) (Sigma) and 100 U/mL RNAse A (Applichem Inc., CT, USA) was added to each sample tubes and incubated for 30 min in the dark at room temperature. Cell cycle was analyzed using Guava PCA flow cytometer with Cytosoft software (Guava technologies Inc, Hayward, CA, USA) as previously described [48].

### 4.6. Induction of Adipogenesis

The induction of differentiation was previously reported [15]. Briefly, 3T3-L1 preadipocytes were seeded at 2.5 × 10^4^ cells/mL in six-well plates and were allowed to adhere overnight. A schedule of media change and hormone additions [0.5 μM 1-isobutyl-3-methylxanthine (IBMX), 1 μM dexa-methasone (DEX), 10 µg/mL insulin] was carried out in a course of 10 days. PGE was added to all media preparations throughout the differentiation process; controls consisted of identical composition but without the addition of PGE. PGE was added at the concentrations of 1 and 10 μg/mL respectively. On Day 2, media was replaced with initiation media (0.5 mM IBMX and 1 μM DEX). On Day 4, media was replaced with progression media (10 µg/mL insulin). On Day 6 and Day 8, fresh DMEM media was added without the additional hormones. Cells were harvested on Day 10 for Oil Red O staining and triglycerides quantification (described below). 

### 4.7. Oil Red O Staining and Quantification of Triglycerides

Oil Red O staining of the lipid acquisition was conducted. Media was removed and set aside while cells were rinsed twice with PBS, and incubated with 10% formalin in PBS for 1 h. Cells were rinsed with deionized water (DI) and then incubated with 2 mL/well Oil Red O solution (0.3% w/v in 60% isopropanol) for 1 h. Plates were again rinsed with DI water, allowed to dry and sent for imaging. Images of stained lipid droplets were captured using Olympus BX51 (U-25ND25-2) microscope with imaging software (Center Valley, PA, USA). 

Triglyceride levels in media were quantified using commercial triglyceride kit (Wako Pure Chemical, Osaka, Japan), as an indication of the lipolytic activity of the cells [17]. According to the manufacturer, 0.5 mL of the media was incubated with 1 mL of the triglyceride quantification reagent and left in the dark at 37 °C for 5 min. Absorbance was measured at 600 nm with 700 nm as reference wavelength. Fresh DMEM was used as blank control to minimize interference that may be potentially caused by triglyceride that is originally present in the media. A standard curve was plotted using the standard provided within the range of 3.1 μg/mL to 15.6 μg/mL.

### 4.8. Adipocyte Viability and Western Blot Analysis

Adipocyte viability was assessed using Guava flow cytometry system with CytoSoft software containing ViaCount module (Guava Technologies) at the end of the differentiation period. 3T3-L1 cells were seeded in six-well plates as described above. Controls consisted of test model cells and DMEM but without the addition of PGE, while treated cells were added with PGE at the concentrations of 1 and 10 μg/mL respectively. On Day 10, adherent cells were rinsed with PBS and were collected by trypsinization (described above) and suspended in equal volume of DMEM. An aliquot of 20 μL from cells media was added to 380 μL of Guava ViaCount reagent and incubated for 5 min in 1.5 mL microcentrifuge tubes prior to data acquisition. 

Adipogenesis was induced in 3T3-L1 cells under the conditions described above. Rabbit polyclonal primary antibodies β-Actin, rabbit polyclonal anti-adiponectin (Acrp30) were purchased from Abcam (Cambridge, UK), and goat polyclonal secondary antibody to rabbit IgG – H&L horseradish peroxidase (HRP) were purchased from BST Scientific (Singapore). Upon completion, adipocytes were lysed with cell lytic reagent (Sigma) and centrifuged at 16160 g for 15 min. The supernatants were collected for β-actin and adiponectin (Acrp30) western blot analysis. According to manufacturer’s instructions, protein extracts were quantified using BCA method (Bio-Rad Laboratories, Hercules, CA, USA) and measured by a spectrophotometer at an absorbance of 595 nm prior to western blot analysis. The protein extract (30 μg per lane) was separated using sodium dodecyl sulfate – polyacrylamide gel electrophoresis (SDS-PAGE) on 15% polyacrylamide separating gel (Mini-Protean Tetra Cell, Bio-Rad Laboratories). The separated protein on gel was then transferred onto a nitrocellulose (NC) membrane (ClearPAGE, C.B.S. Scientific, Del Mar, CA, USA) by a semi-dry transfer blotter (C.B.S. Scientific). NC membranes were first incubated with 5% skim milk in phosphate-buffered saline with 0.1% Tween-20 (TBST) for 1 h at room temperature to increase specificity in antibodies binding. Membranes were then separately incubated with diluted primary antibody (β-actin, 1:1,000, Acrp30, 1:250) blotted with 5% skim milk in TBST and were gently agitated overnight at 4 °C. On the following day, membranes were washed three times with TBST for 5 min, followed by incubating with secondary antibodies (HRP) (1:10,000) in 5% skim milk in TBST for 1 h, and washed three times with TBST for 5 min each. Enhanced chemiluminescence (Thermo Scientific) was added onto the NC membranes and band intensities of β-Actin and Acrp30 were visualized using a Fluorchem FC2 Imaging System (Alpha Innotech, San Leandro, CA, USA). Protein expression was quantified densitometrically using the software GelPro 32 (Media Cybernetics, Bethesda, MD, USA) and expressed in relative to β-actin as reference bands. 

### 4.9. Statistical Methods

The experimental replications were as follows: plant materials were extracted three times and analyzed (HPLC-MS). MTT assay utilized eight replicates in three separate experiments. Cell cycle, oil red O staining, triglycerides and protein quantification were performed in triplicates in three separate experiments. Cell ViaCount analysis included three individual experiments with duplicates and western blot analysis was performed on three different cell lysates repeated in three separate experiments on different days. Data were expressed as mean ± standard deviation (SD) and were analyzed using ANOVA (HPLC, cell cycle, triglycerides, Western blot) and paired T-test (ViaCount) to compare control with PGE. Differences were considered significant when compared to control at *P <* 0.05. 

## 5. Conclusions

We have shown that *Panax ginseng* extract (PGE) with a known amount of ginsenosides is capable of suppressing preadipocyte growth, reducing lipid acquisition and triglycerides, while increasing adiponectin expression in 3T3-L1 cells. Further studies are needed to elucidate the exact mechanisms responsible for the lipid-suppressing activity of specific ginsenosides or combinations of these within extracts.
